# Intermittent Preventive Treatment of Malaria in Pregnancy: Assessment of the Sulfadoxine-Pyrimethamine Three-Dose Policy on Birth Outcomes in Rural Northern Ghana

**DOI:** 10.1155/2019/6712685

**Published:** 2019-06-02

**Authors:** Francis Anto, Ibrahim Haruna Agongo, Victor Asoala, Elizabeth Awini, Abraham Rexford Oduro

**Affiliations:** ^1^School of Public Health, University of Ghana, Legon, P.O. Box LG 13, Accra, Ghana; ^2^Navrongo War Memorial Hospital, Navrongo, P.O. Box 34, UE/R, Ghana; ^3^Navrongo Health Research Centre, Navrongo, P.O. Box 114, UE/R, Ghana; ^4^Dodowa Health Research Centre, P.O. Box DD 1, Dodowa, GA/R, Ghana

## Abstract

**Background:**

Intermittent preventive treatment of malaria in pregnancy with sulfadoxine-pyrimethamine (IPTp-SP) decreases placental parasitaemia and improves birth outcomes. Currently, WHO recommends three or more doses of SP given during antenatal care (ANC), spaced one month apart after 16 weeks of gestation till delivery. This study determined the level of uptake of SP and its association with birth outcomes in rural northern Ghana.

**Methods:**

A survey was carried out at the War Memorial Hospital in Navrongo, Ghana, among mothers who had delivered within ten weeks and were seeking postnatal care. Data on time of first ANC, number of visits, receipt of IPTp-SP, and birth outcomes were extracted from the antenatal records of 254 mothers. Mothers were interviewed on their background characteristics and obstetric history. Chi-square tests and logistic regression were carried out to determine association between antenatal indicators, uptake of IPTp-SP, and birth outcomes using Stata version 13.

**Results:**

Uptake of three-five doses of SP was IPT3 =76.4%, IPT4 =37.3%, and IPT5 = 16.0%. Receipt of first dose of SP at 16, 17-24, and 25-36 weeks of gestation was 16.9%, 56.7%, and 26.4%, respectively. Taking the first dose of SP during the second trimester allowed for taking ≥3 doses of SP compared to taking the first dose during the third trimester (*χ*2 = 60.1,* p*<0.001). Women who made ≥4 visits were more likely to receive ≥3 doses of SP compared to those who made <4 visits (*χ*2 = 87.6,* p*<0.001). Women who received ≥ 3 doses of SP were more likely (OR = 3.3; 95% CI: 1.69-6.33) to give birth at term and also have normal weight babies (OR =4.0; 95% CI: 1.98-8.06).

**Conclusion:**

Uptake of three or more doses of SP contributed to improved pregnancy outcomes. Increased efforts towards improving early ANC attendance could increase uptake of SP and improve pregnancy outcomes.

## 1. Background 

Malaria is a life-threatening disease caused by* Plasmodium* parasites and exerts the highest burden in Africa south of the Sahara [1]. Although the disease is preventable and curable and is currently receiving global attention, it is still a disease of public health significance with an estimated 219 million cases and 435,000 deaths globally in 2017. Most of these deaths (93%) occurred in Africa [2].

Pregnant women and children under five years bear the brunt of the disease with over 70% of all malaria deaths being reported among children under five years. Malaria infection during pregnancy is of public health concern as it poses significant risk in terms of morbidity and mortality to both the pregnant woman and the developing foetus. Malaria-associated maternal morbidity and poor birth outcomes including preterm delivery and low birth weight are due primarily to* P. falciparum* infection and occur mostly in Africa [3].

Intermittent preventive treatment of malaria in pregnancy (IPTp) using sulfadoxine-pyrimethamine (SP) remains an effective strategy for preventing malaria in pregnancy. This strategy entails the administration of a full treatment dose of SP to pregnant women when they visit antenatal care (ANC) facilities for health services, regardless of whether the pregnant woman has malaria infection or not [3]. The IPTp-SP strategy is effective in reducing the level of malaria infection in pregnancy, maternal anaemia, low birth weight, and neonatal mortality.

The World Health Organization (WHO) revised the recommendations for IPTp-SP in 2012 and now calls for all pregnant women to take SP at each ANC visit until delivery. The administration of SP should start early in the second trimester, with doses taken at least one month apart [4]. The recommendations further call for all pregnant women in areas with moderate to high malaria transmission in Africa to receive at least three doses during each pregnancy. Reports have shown that the number of ANC visits made by the pregnant woman and early uptake of the first dose of SP are major determinants of uptake of higher doses of IPTp-SP [5, 6] and therefore the benefits derived from the programme.

The Malaria Control Programme of Ghana also revised the national policy and now requires every pregnant woman to take a minimum of five doses of SP during each pregnancy [7]. The SP is to be taken at monthly intervals as they attend antenatal clinics starting from 16 weeks of gestation until delivery. The new policy became effective in the country in 2014. A recent hospital-based study in the capital city, Accra, revealed a low (14%) level of uptake of five doses of SP [6].

The purpose of the current study was to assess the level of uptake of more than three doses of SP and its relationship with birth outcomes at the Navrongo War Memorial Hospital, a rural district hospital in northern Ghana.

## 2. Methods

### 2.1. Study Area

The study was carried out at the War Memorial Hospital (WMH) at Navrongo, the capital of the Kassena-Nankana East Municipality (KNEM) of northern Ghana. The KNEM lies in the Sahelian savannah between latitude 10°30' and 11°00' north and longitude 1°00' and 1°30' west and covers an area of about 1,674 square kilometres of land with a population of about 110,000 [8]. The area is characterized by two distinct seasons: a rainy season from June to October and a hot dry season from November to May. Annual rainfall averages 850 mm with mean daily temperature ranges of 20°C to 40°C. Malaria transmission is perennial with distinct seasonal patterns. Prevalence of infection is highest during the rainy season and lowest during the dry season [9]. Malaria infection is due mainly to* Plasmodium falciparum *with* Anopheles gambiae *and* An. funestus *being the main vectors with an annual entomological inoculation rate of 418 [10].

The WMH has a total bed capacity of 169 and is the only hospital in the municipality and serves as a referral centre for all other health facilities in the municipality and adjoining districts including Bulsa and Sissala West.

### 2.2. Study Design

A cross-sectional study was carried out at the postnatal and child welfare clinics of the Navrongo War Memorial Hospital in northern Ghana. Nursing mothers who had delivered within the past 10 weeks were enrolled into the study. These mothers were recruited on a daily basis in a sequential manner as they reported at the units for care. Data on demographic characteristics, number of ANC visits, and uptake of IPTp-SP were collected from the mothers onto a case record form. The antenatal care cards of the mothers were also reviewed and data on obstetric history and care extracted. Data collection lasted for 13 weeks during the months of June to September 2017.

### 2.3. Sample Size Estimation

The sample size was estimated using the Cochran formula, n= (Z^2^pq)/d^2^, where n is sample size, Z is the z-score that corresponds with the 95% confidence interval (1.96), P is proportion of antenatal attendants who received IPT 3 in 2016 (20.3%, = 0.203), q is proportion of antenatal attendants who received less than three doses of SP (1-0.203, = 0.797), and d is margin of error set at 5% (0.05) [11, 12]. The estimated sample size, n, was 249.

### 2.4. Inclusion/Exclusion Criteria

All nursing mothers who visited the postnatal or child welfare clinics of the WMH for healthcare during the study period and gave written informed consent to participate in the study were eligible to participate. Nursing mothers who had delivered beyond ten weeks at the time of data collection were excluded to minimize recall bias.

### 2.5. Data Collection Procedure

One-on-one interviews were held with the mothers and data on background characteristics including age, education, number of children, occupation, and marital status collected from them onto a case record form designed specifically for this study. The ANC cards of the mothers were also reviewed and information on gestational age at first ANC visit, number of visits, number of doses of SP taken before delivery, and the gestational age at which the first and subsequent doses of SP were taken was extracted. Other data extracted from the ANC card included malaria infection during the most recent pregnancy and the method of confirmation (RDT or microscopy), gestational age of delivery, birth weight, and length of baby. Whenever there was discrepancy between the information given by the mother and what was documented, the information on the ANC card was used. The interviews were conducted in the local languages (Kassem, Nankam, and Buli) and English by trained research assistants (midwives), who are fluent in these languages. The questionnaire was in English and so the information was recorded in English.

### 2.6. Quality Control

The questionnaire was pretested using 20 ANC attendants over a period of two days (10 per day). The pretest data collected were analysed to inform the content and formatting of the final questionnaire that was used for the data collection. The pretest was conducted one week before commencement of the actual data collection at the same facility, as this is the only hospital in the municipality. The data were however not included in the study. The midwives who did the data collection were trained for three days on how to obtain informed consent, explain the objectives of the study to participants, and complete the questionnaire.

### 2.7. Data Processing and Analysis

Data were entered into Excel version 2013, cleaned, and imported to Stata version 13 for analysis. The uptake of IPTp-SP was categorised into < 3 and ≥ 3 doses. The sociodemographic and ANC characteristics were also grouped into categories. The birth weight of the babies was categorised into low birth weight (< 2.5 kg) and normal birth weight (≥ 2.5 kg). Time of delivery was categorised into preterm (< 37 weeks' gestation) and term (37-42 weeks). The length of the babies was categorised into < 45.7 cm and ≥ 45.7 cm. Bivariate analysis was done using Pearson chi-square tests to assess significant association between IPTp-SP uptake and each independent categorical variable. Factors with* p* value < 0.05 at 95% CI were considered statistically significant. Since the study examined the effect of ≥ 3 doses of IPTp-SP on the various birth outcomes, logistic models were fitted separately for each of the outcomes (gestational age at delivery, delivery outcome, birth weight, length of baby, and baby head circumference), adjusting for other maternal factors in each of the models. The factors adjusted for included ITN use, educational status, number of previous children, and malaria infection during pregnancy.

## 3. Results

### 3.1. Background Characteristics of Study Mothers

Two hundred and fifty-four nursing mothers took part in the study. Their ages ranged from 15 to 47 years (median: 26 years; IQR: 21-28), 56.3% (143/254) were aged 20-29 years, and 85.8% were married. One hundred and nine (42.9%) of the mothers had formal education up to the secondary level, with 11.0% without formal education. The majority of the participants (57.5%, 146/254) were engaged in some form of self-employment including farming, with 27.0% of them practicing a trade, including dressmaking and hairdressing. Most of the mothers (64.6%, 164/254) had one or two children with an average number of two children (range: 1-6) (Table 1).

### 3.2. ANC Attendance, ITN Use, IPTp-SP Uptake, and Malaria Infection

**Figure 1:**
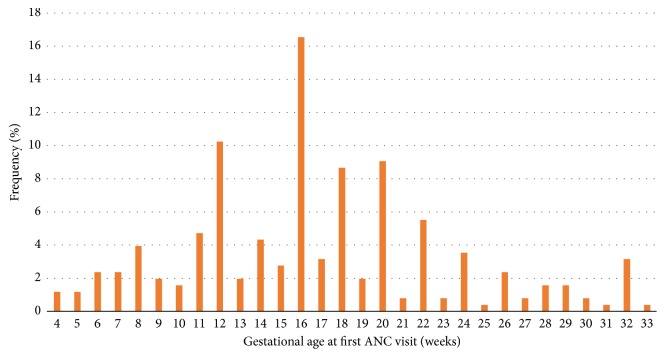
Gestational age at first ANC visit among women that had recently delivered at the Navrongo War Memorial Hospital in rural northern Ghana.

Only 75 (29.5%) out of the two hundred and fifty-four mothers made their first ANC visit during the first trimester of their pregnancy, with 59.1% making the first visit during the second trimester. The mean gestational age at the time of the first ANC visit was 16.7 weeks (SD: 6.43; range: 4-33 weeks). The number of ANC visits made ranged from 0 to 14 (mean: 7.5; SD: 8.57), with two of the mothers (0.8%) not making any ANC visits during their most recent pregnancy. A total of 109 (42.9%) of the mothers made five to seven visits, and 93 (36.6%) made eight or more visits before delivery (Table 2). Most of the mothers made their first ANC visit during weeks 12, 16, 18, and 20, with fewer numbers visiting during the other gestational weeks (Figure 1). Forty-three (16.9%) of the mothers received the first dose of SP at 16 weeks of gestation with 56.7% (144/254) receiving the first dose during the period of 17-24 weeks of gestation. ITN usage was found to be high with most (95.3%, 242/254) of the mothers indicating that they slept under an ITN the night before the survey and 93.7% (238/254) indicating that they slept under ITN most of the time during the most recent pregnancy. Malaria infection during the most recent pregnancy was found to be low (7.9%, 20/254) (Table 2).

### 3.3. Gestational Age at First Dose of IPTp-SP

**Figure 2:**
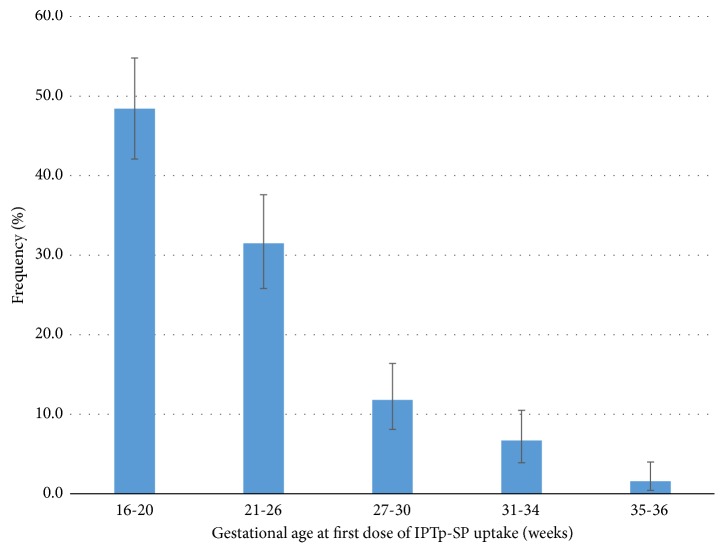
Gestational age at first dose of IPTp-SP uptake among women that had recently delivered at the Navrongo War Memorial Hospital in rural northern Ghana. The points plotted (-) indicate the percentage of mothers who took the first dose at the particular gestational age, while the vertical lines show the corresponding 95% confidence intervals.

Twenty-nine (11.4%) of the mothers received only one dose of SP during their most recent pregnancy. Ninety-nine (39.0%) received three doses; IPTp-SP coverage of at least three doses was 76.4% (194/254). Forty-one of the mothers received five or more doses of SP giving coverage of ≥ 5 doses of 16.1% (Table 2). The median gestational age at which respondents received the first dose of SP was 21 weeks (IQR: 18-25) ranging from 16 to 36 weeks. Sixty-three (24.8%) of the mothers received the first dose of SP on their first ANC visit, with most of the mothers receiving the first dose during weeks 16-26. Thus by the end of the WHO recommended third contact period, 79.9% (203/254) of the mothers had received their first dose of SP, with fewer numbers receiving their first SP dose during the other contact periods (Figure 2).

The gestational age at which the first ANC visit was made, the total number of ANC visits that were made, and the gestational age at which the first dose of SP was received were found to be significantly associated with the total number of doses of SP received before delivery (*p*<0.001). The educational level of the mother was also found to be associated with uptake of SP (*χ*^2^ = 8.8;* p*<0.05). Other factors like number of children that a mother had, her marital status, and age were not associated with SP uptake (*p*>0.05) (Table 3).

The difference between mothers making their first ANC visit during the first trimester and those making the first visit during the second trimester in terms of receiving ≥ 3 doses of SP was not statistically significant (*p*>0.05). Four or more ANC visits however offered the opportunity for mothers to receive ≥ 3 doses of SP compared to those visiting the ANC < 4 times (*χ*^2^ = 87.6,* p*<0.001) (Table 3).

Mothers who also took their first dose of SP during week 16 of pregnancy were able to receive ≥ 3 doses compared to those who took the first dose during weeks 25-36 (*χ*^2^ = 24.30,* p*<0.001) (Table 3). Similarly, mothers who took their first dose during weeks 17-24 were more likely to receive ≥ 3 doses compared to those who received the first dose during weeks 25-36 (*χ*^2^ = 48.15,* p*<0.001). Taking the first dose in week 16 however did not make a difference in terms of a mother being able to receive ≥ 3 doses compared to taking it during any of weeks 17-24 (*p* > 0.05).

### 3.4. IPTp-SP Uptake by Mothers and Background Characteristics of Babies Delivered

A total of 247 (97.2%) live babies were delivered, with most of them (77.6%, 197/254) delivered at term (37-42 weeks). Two hundred and nine (82.3%) of the babies weighed ≥ 2.5 kg with mean birth weight of 2.9 kg (SD: 0.54; range: 2.0-4.3 kg). Most of the babies (83.1%) were ≥ 45.7 cm in length at birth (mean: 49.0 cm; SD: 4.0; range: 33-61 cm). The majority of the babies (83.7%, 210/254) had head circumference < 35 cm (mean: 32.0 cm; SD: 6.32; range: 21-39 cm) (Table 4).

Statistically significant association was found between doses of SP taken by mothers, gestational age at delivery, and birth weight of babies (*p*<0.05). No association was found between doses of SP, live birth, length of baby, and head circumference of baby at birth. Logistic regression analysis established that women who received ≥ 3 doses of SP were more likely (AOR = 3.16; 95% CI: 1.36-5.42) to give birth at term and also have normal weight babies (AOR =3.92; 95% CI: 1.94-9.92). Although uptake of ≥ 3 doses of SP was not protective against malaria, having malaria during pregnancy was more likely (3.78; 95% CI: 1.36-10.47) to result in preterm delivery (Tables 5(a) and 5(b)). Logistic regression analysis of other birth outcomes is included in Additional File 3.

## 4. Discussion

Intermittent preventive treatment of malaria in pregnancy using SP (IPTp-SP) is known to reduce maternal malaria episodes and improve pregnancy outcomes [3]. Uptake of higher doses of the drug before delivery significantly increases the benefits. To the best of our knowledge, the current paper is the first report from the KNEM of northern Ghana since the revision of the WHO policy on IPTp-SP in 2012. The current report has established that even though the majority of the women including those who delivered preterm made at least four ANC visits as recommended in the previous WHO policy, only 37% of them made the required eight visits per the new policy [13]. Most of these women (76%) received three or more doses of SP as recommended in the new WHO policy, but only 16% received five doses of SP in line with the new Ghana policy. Uptake of three or more doses of SP was associated with delivery at term and having normal birth weight babies.

Antenatal care is very important in the prevention, early detection, and treatment of general medical and pregnancy-related conditions. All pregnant women are therefore expected to go for their first ANC visit in the first trimester [13], to allow for early diagnosis and manage of any medical conditions as well as to screen for any risk factors that could affect the progress and outcome of the pregnancy. The results of this study in rural northern Ghana show that less than 30% of the mothers made their first ANC visit during the first trimester which is much lower than the over 40% reported in an earlier study in Accra, the capital city of Ghana [6]. This is also lower than reports from some other African countries including South Africa [14], Cameroon [15], and the Democratic Republic of Congo [16]. This proportion of women who made their first ANC visit during the first trimester is also much lower than that reported in an earlier study conducted in another district in northern Ghana [17] but similar to results from Njim and colleagues' study in Cameroon [18].

There are reports that initiation of ANC visits in Africa is generally late with most women making their first visit during the second trimester, with significant variations across subregions and settings [18–20]. Several factors, including employment, marital and financial status, planning for the particular pregnancy, educational level of the woman, and distance to the hospital, have been reported to influence time of first ANC visit [14–16, 19]. Addressing some of these economic and social problems can help reduce the prevalence of late initiation of ANC visits. Late initiation of ANC visits can reduce the intended benefits of the service for pregnant women and increase the risk of poor pregnancy outcomes as well as maternal and neonatal mortality.

In our current study, no significant association was observed between marital status and number of doses of SP taken even though a higher proportion of married women took the drug compared to the single or divorced women. The level of formal education was found to be important in the uptake of more doses of SP. Higher education is likely to improve on one's understanding of the benefits of the policy and therefore willingness to take full advantage of it. This supports the earlier report by Nsibu and colleagues from the Democratic Republic of Congo that pregnant women living alone or without much education are likely to initiate ANC late [16] and therefore receive fewer doses of SP. Single or divorced women may not get the moral and financial support needed from husbands to travel to the ANC centres.

Since normally ANC services are sought on a monthly basis, starting late will not allow for achieving the recommended eight visits [13]. In the current study therefore, only 37% of the mothers were able to make the required eight ANC visits. This also affected the number of SP doses received [17], as early ANC visit enables the uptake of more doses of SP [21, 22]. Even though most of the women received the WHO recommended number of SP doses of three or more, which was the requirement of the old Ghana malaria control programme policy, only 16% of them received the five doses recommended by the new Ghana policy. The proportion of women receiving five doses of SP in rural northern Ghana was similar to that reported for the capital city, Accra [6], possibly as a result of late initiation of ANC.

The prevalence of low birth weight as found in the current study seems not to have changed since an earlier report from the same health facility by Oduro and colleagues eight years ago [23]. Intermittent preventive treatment of malaria in pregnancy using sulfadoxine-pyrimethamine (IPTp*-*SP) is however known to be effective in reducing maternal malaria episodes, low birth weight, and preterm delivery [21, 24, 25]. These benefits have all been linked to the number of doses of SP taken by the pregnant woman [21] before delivery. Uptake of more doses of SP reduces the prevalence and intensity of placental malaria, and placental parasitaemia is known to contribute significantly to having preterm delivery and low birth weight infants [26]. Uptake of three or more doses of SP in the current study therefore was found to be related to having normal birth weight babies and delivery of babies at term. Our current study did not demonstrate the beneficial effect of higher doses SP being able to protect pregnant women against episodes of malaria, possibly because most of the pregnancies were during the dry season when malaria transmission was low, with only 20 cases of malaria. Further analyses however revealed that episodes of malaria were associated with preterm delivery.

The study has some limitations. First, information was collected from mothers receiving postnatal care in a rural community on their ANC attendance and services provided to them by the healthcare staff during their most recent pregnancy. There is a possibility of recall bias; however, recall was limited to only three months which might not affect the reliability of responses given. Also, the ANC cards were reviewed and information collected from the mothers was validated. Second, it is important to note that even as receiving ≥ 3 doses of IPTp-SP significantly influenced delivery at term, delivery at preterm could affect the number of doses received before delivery. The mean and median number of times of ANC visits by mothers who delivered preterm was five, which was more than the recommended minimum of four visits.

### 4.1. Conclusion

The uptake of ≥ 3 doses of IPTp-SP was quite high in this rural community but much lower than in an earlier study in Accra. The proportion of mothers who received five doses of SP as recommended by the new Ghana policy was very low but similar to the earlier report from Accra. Uptake of higher doses of SP was significantly associated with delivery at term and normal birth weight babies. Equal attention would be needed in both rural and urban communities in Ghana to increase uptake of SP and improve pregnancy outcomes in order to achieve sustainable development goal three. It is therefore recommended that healthcare providers especially midwives should encourage women in their fertility age to seek early ANC service when they are pregnant and have regular monthly visits to increase uptake of SP and therefore better pregnancy outcome. In the long term, formal education for the girl child should be encouraged to improve knowledge and birth outcome.

## Figures and Tables

**Table 1:** Background characteristics of study mothers.

Characteristics	No.	%
*Age (years)*		
15-19	35	13.8
20-29	143	56.3
30-39	67	26.4
40-47	9	3.5
*Marital status*		
Married	235	85.8
Single	37	13.4
Divorced	2	0.8
*Educational level*		
No formal education	28	11.0
Basic education	61	24.0
Secondary education	109	42.9
Tertiary education	56	22.1
*Occupation*		
Trading	54	21.2
Housewife	56	21.5
Farming	24	9.5
Civil service	52	20.8
Practicing a trade	68	27.0
*Number of children alive including current child *		
1-2	164	64.6
3-4	75	29.5
5-6	15	5.9

**Table 2:** ANC attendance, ITN use, IPTp-SP uptake, and malaria infection during current pregnancy by mothers.

Characteristics	No.	%
*Gestational age at first ANC*		
First trimester	75	29.5
Second trimester	150	59.1
Third trimester	29	11.4
*Number of ANC visits*		
No ANC visit	2	0.8
1-4	50	19.7
5-7	109	42.9
≥ 8	93	36.6
*Number of IPTp-SP doses received*		
One	29	11.4
Two	31	12.2
Three	99	39.0
Four	54	21.3
Five	37	14.6
Six	4	1.6
*Gestational age at first dose of SP (weeks) *		
16	43	16.9
17-24	144	56.7
25-36	67	26.4
*SP taken under DOT*		
All the time	247	97.2
Most of the time	7	2.8
*Use of ITN the previous night*		
Used ITN	242	95.3
Did not use ITN	12	4.7
*Malaria infection during current pregnancy*		
Had infection	20	7.9
Did not have infection	234	92.1

ANC= antenatal centre, ITN = insecticide treated net, SP = sulfadoxine pyrimethamine, and IPTp = intermittent preventive treatment in pregnancy.

**Table 3:** Relationship between ANC visits, sociodemographic characteristics, IPTp-SP uptake, and malaria infection among women that had recently delivered.

Variables	No.	% uptake of IPTp-SP		*χ*2	*p* value
		< 3 doses	≥ 3 doses		
*Gestational age at first ANC*					
First trimester	75	12.0	88.0	51.0	<0.001
Second trimester	150	19.3	80.7		
Third trimester	29	75.9	24.1		
*Number of ANC visits*					
< 4	29	93.1	6.9	87.6	<0.001
≥ 4	225	57.8	42.2		
*Gestational age at first dose of SP*					
16 weeks	43	9.3	90.7	60.5	<0.001
17-24 weeks	144	11.8	88.2		
25-36 weeks	67	58.2	41.8		
*Number of children *					
1-2	164	24.4	75.6	1.4	
3-4	75	20.0	80.0		0.501
5-6	15	33.3	66.7		
*Marital status*					
Married	218	21.6	78.4	3.6	0.146
Single	34	35.3	64.7		
Divorced	2	50	50		
*Educational level*					
No formal education	28	42.9	57.1	8.8	0.032
Basic education	61	26.2	73.8		
Secondary education	109	22.0	78.0		
Tertiary education	56	14.3	85.7		
*Age group (years)*					
15-19	35	37.1	62.9	5.0	0.175
20-29	143	20.3	79.7		
30-39	67	22.4	77.6		
40-47	9	33.3	66.7		
*Malaria infection during current pregnancy*					
Had infection	20	20.0	80.0	0.16	0.691
Did not have infection	234	23.9	76.1		

IPTp-SP= intermittent preventive treatment in pregnancy with sulfadoxine pyrimethamine, n= number of respondents.

**Table 4:** IPTp-SP uptake, gestation at delivery, outcome of delivery, and anthropometric indices of babies.

Characteristics	No.	%	% uptake of IPTp-SP		*χ*2	*p* value
*Gestation age at delivery (weeks)*			< 3 doses	≥ 3 doses		
< 37	57	22.4	40.4	59.6	11.4	0.001
37-42	197	77.6	18.8	81.2		
*Outcome of delivery *						
Alive	247	97.2	23.1	76.9	1.4	0.224
Dead	7	2.8	42.9	57.1		
*Birth weight (kg)*						
< 2.5	45	17.7	48.9	51.1	19.4	<0.001
≥ 2.5	209	82.3	18.2	81.8		
*Length of baby at delivery (cm)*						
< 45.7	43	16.9	33.3	66.7	2.6	0.105
≥ 45.7	211	83.1	21.7	78.3		
*Head circumference (cm)*						
< 35.0	210	83.7	23.8	76.2	0.02	0.878
≥ 35.0	44	17.3	23.6	76.4		

IPTp-SP= intermittent preventive treatment in pregnancy with sulfadoxine pyrimethamine.

**Table:** (a) Crude and adjusted associations between gestation at delivery (weeks) and uptake of ≥3 doses of SP

Characteristics	Crude OR	95% CI	*p* value	Adjusted OR	95% CI	*p* value
*SP doses*						
< 3 doses of SP	1.00			1.00		
≥3 doses of SP	2.93	1.54-5.54	0.001	3.16	1.63-6.11	0.001
*Used ITN*						
Used ITN	1.00			1.00		
Did not use	0.87	0.23-3.31	0.834	1.25	0.27-5.82	0.773
*Maternal education*						
No formal education	1.00			1.00		
Primary education	1.66	0.80-3.41	0.171	1.83	0.84- 4.00	0.128
JHS and above	1.65	0.77-3.55	0.200	1.71	0.76-3.81	0.193
*Number of children*						
<3	1.00			1.00		
≥3	1.25	0.66-2.34	0.490	1.33	0. 68-2.62	0.401
*Malaria pregnancy *						
Had malaria	1.00			1.00		
Did not	3.12	1.22-7.96	0.017	3.78	1.36-10.47	0.011

**Table:** (b) Crude and adjusted associations between birth weight (kg) and uptake of ≥3 doses of SP

Characteristics	Crude OR	95% CI	*p* value	Adjusted OR	95% CI	*p* value
*SP doses*						
< 3 doses of SP	1.00			1.00		
≥3 doses of SP	4.30	2.18-8.51	<0.001	3.92	1.94-9.92	<0.001
*Used ITN*						
Used ITN	1.00			1.00		
Did not use	1.09	0.23-5.13	0.919	1.05	0.19-5.89	0.955
*Maternal education*						
No formal education	1.00			1.00		
Primary education	1.21	0.55- 2.63	0.640	1.31	0.56-3.05	0.535
JHS and above	1.63	0.68-3.86	0.272	1.63	0.66-4.01	0.291
*Number of children*						
<3	1.00			1.00		
≥3	1.12	0. 57-2.21	0.746	1.04	0.50 -2.16	0.924
*Malaria pregnancy*						
Had malaria	1.00			1.00		
Did not	1.67	0.58-4.94	0.353	1.88	0.59-6.04	0.288

## Data Availability

All data generated during the current study are included in this published article and its supplementary information file (Additional files 1 and 2).

## Abbreviations

ANC: Antenatal care
CWC: Child welfare clinic
DOT: Directly observed therapy
IPT: Intermittent preventive treatment
IPTp-SP: Intermittent preventive treatment of malaria in pregnancy with sulfadoxine pyrimethamine
KNEM: Kassena-Nankana East Municipality
NMCP: National Malaria Control Programme
PMI: President's Malaria Initiative
SP: Sulfadoxine pyrimethamine
WMH: War Memorial Hospital.

## Supplementary Materials

Supplementary Materials Additional File 1: dataset. Additional File 2: Table 6: List of variables measured. Additional File 3: Table 7: Logistic regression analysis II.
