# Supplementary material for: Intermittent Preventive Treatment of Malaria in Pregnancy: Assessment of the Sulfadoxine-Pyrimethamine Three-Dose Policy on Birth Outcomes in Rural Northern Ghana
Source: J Trop Med. 2019 Jun 2;2019:6712685. doi: 10.1155/2019/6712685 (PMC6582795; doi:10.1155/2019/6712685)
Supplement: Supplementary Materials — Additional File 1: dataset. Additional File 2: Table 6: List of variables measured. Additional File 3: Table 7: Logistic regression analysis II. [file 6712685.f1.zip › Additional file 2.docx]

**Table 6. List of variables measured**

| ***Variables*** | ***Operational definitions*** | ***Type of variable*** |
| --- | --- | --- |
| **Primary outcome** |  |  |
| Uptake of IPTp-SP | Doses of SP received during pregnancy | Binary Variable |
| **Secondary outcomes** |  |  |
| Live birth | Born dead or alive | Binary Variable |
| Birth weight | Weight of baby in kg as recorded by the midwife | Continuous Variable |
| Length at birth | Length of baby in cm as recorded by the midwife | Continuous Variable |
| Gestational age at delivery | Stage of pregnancy at delivery in weeks as recorded in ANC book | Continuous Variable |
| **Independent** |  |  |
| *Socio-demographic, characteristics* |  |  |
| Age | The age in years of the woman | Continuous Variable |
| Marital Status | Married, single or divorced | Categorical Variable |
| Level of Education | Stage of education attained | Categorical Variable |
| Occupation | Self Employed or Government employed or unemployed | Categorical Variable |
| Number of children | The number of live births of the woman | Continuous variable |
| Number of ANC visits | The number of visits to ANC during last pregnancy | Continuous Variable |
| Gestational age at first dose of SP | Stage of pregnancy in weeks at receiving first dose of SP | Continuous Variable |
| Gestational age at first ANC visit | Stage of pregnancy in weeks at first ANC visit | Continuous Variable |
