# Supplementary material for: Intermittent Preventive Treatment of Malaria in Pregnancy: Assessment of the Sulfadoxine-Pyrimethamine Three-Dose Policy on Birth Outcomes in Rural Northern Ghana
Source: J Trop Med. 2019 Jun 2;2019:6712685. doi: 10.1155/2019/6712685 (PMC6582795; doi:10.1155/2019/6712685)
Supplement: Supplementary Materials — Additional File 1: dataset. Additional File 2: Table 6: List of variables measured. Additional File 3: Table 7: Logistic regression analysis II. [file 6712685.f1.zip › Additional file 3.docx]

**Logistic regression analysis II**

**Table 7a: Crude and adjusted associations between Outcome of delivery and uptake of ≥3 doses of SP**

| Characteristics | Crude OR | 95% CI | p-value | Adjusted OR | 95% CI | p-value |
| --- | --- | --- | --- | --- | --- | --- |
| SP doses  < 3 doses of SP  ≥3 *doses of SP* | 1.00  2.5 | 0.54-11.5 | 0.239 | 1.00  2.60 | 0.56-12.0 | 0.225 |
| Maternal Education  No formal education  Primary  JHS and above | 1.00  0.44  0.68 | 0.05-4.00  0.06-7.71 | 0.464  0.758 | 1.00  0.50  0.72 | 0.05-4.76  0.06-8.30 | 0.548  0.794 |
| Number of children  <3  ≥3 | 1.00  3.38 | 0.40-28.5 | 0.263 | 1.00  3.99 | 0. 35-25.89 | 0.319 |

**Table 7b: Crude and adjusted associations between Length of baby at delivery (cm) and uptake of ≥3 doses of SP**

| Characteristics | Crude OR | 95% CI | p-value | Adjusted OR | 95% CI | p-value |
| --- | --- | --- | --- | --- | --- | --- |
| SP doses  < 3 doses of SP  ≥3 *doses of SP* | 1.00  1.98 | 0. 97-4.01 | 0.059 | 1.00  2.04 | 1.94-4.20 | 0.052 |
| Used ITN  Used ITN  Did not use | 1.00  2.31 | 0. 29-18.4 | 0.426 | 1.00  2.55 | 0.30-21.6 | 0.391 |
| Maternal Education  No formal education  Primary  JHS and above | 1.00  0.77  0.87 | 0.33- 1.81  0.35- 2.15 | 0.551 0.756 | 1.00  0.79  0.85 | 0.32- 1.93  0.34-2.17 | 0.603  0.741 |
| Number of children  <3  ≥3 | 1.00  1.74 | 0. 83-3.65 | 0.746 | 1.00  1.80 | 0.81-3.87 | 0.130 |
| Malaria pregnancy  Had malaria  No malaria | 1.00  0.84 | 0.24- 3.01 | 0.792 | 1.00  0.88 | 0.23-3.33 | 0.848 |

**Table 7c: Crude and adjusted associations between Head circumference (cm) and uptake of ≥3 doses of SP**

| Characteristics | Crude OR | 95% CI | p-value | Adjusted OR | 95% CI | p-value |
| --- | --- | --- | --- | --- | --- | --- |
| SP doses  < 3 doses of SP  ≥3 *doses of SP* | 1.00  1.06 | 0.49-2.30 | 0.878 | 1.00  0.99 | 0.45-2.17 | 0.980 |
| Used ITN  Used ITN  Did not use | 1.00  1.63 | 0.42-6.27 | 0.480 | 1.00  2.04 | 0.48-8.7 | 0.334 |
| Maternal Education  No formal education  Primary  JHS and above | 1.00  1.73  1.96 | 0.69- 4.37  0.76-5.07 | 0.244 0.166 | 1.00  2.11  1.96 | 0.81-5.50  0.75-5.13 | 0.125  0.172 |
| Number of children  <3  ≥3 | 1.00  1.33 | 0. 68-2.58 | 0.405 | 1.00  1.47 | 0.73-2.97 | 0.279 |
| Malaria pregnancy  Had malaria  No malaria | 1.00  2.0 | 0.45-8.95 | 0.365 | 1.00  2.40 | 0.51-11.38 | 0.269 |
